# Supplementary material for: ATR Contributes More Than ATM in Intra-S-Phase Checkpoint Activation after IR, and DNA-PKcs Facilitates Recovery: Evidence for Modular Integration of ATM/ATR/DNA-PKcs Functions
Source: Int J Mol Sci. 2022 Jul 6;23(14):7506. doi: 10.3390/ijms23147506 (PMC9316047; doi:10.3390/ijms23147506)
Supplement: Supplementary file 1 [file ijms-23-07506-s001.zip › ijms-1772430-supplementary.pdf]

Supplementary Material (IJMS-1772430)

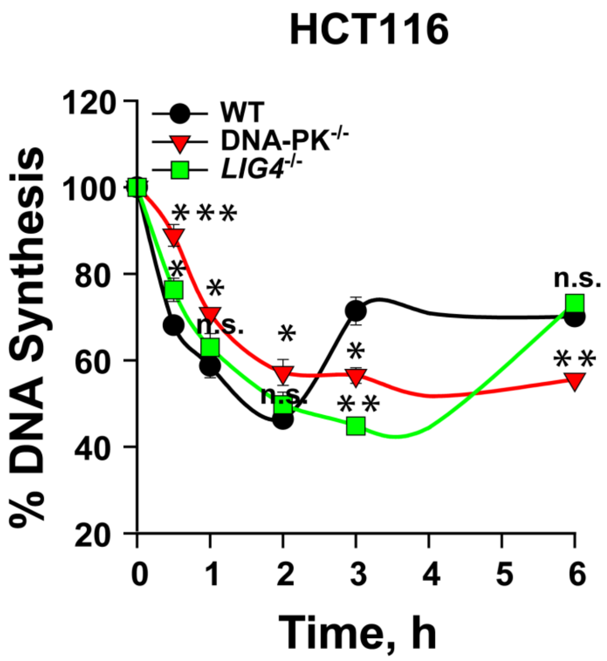

Figure S1 Soni et al.

Figure S1. IR-induced intra-S-phase checkpoint response in human HCT116 WT, DNAPK$^{-/-}$, and *LIG4*$^{-/-}$ cells. Percentage of DNA synthesis over unirradiated controls was measured after exposure to 10 Gy IR. The significance of differences between individual measurements is indicated by * symbol: *$p < 0.05$, **$p < 0.01$, ***$p < 0.001$, n.s. non-significant.

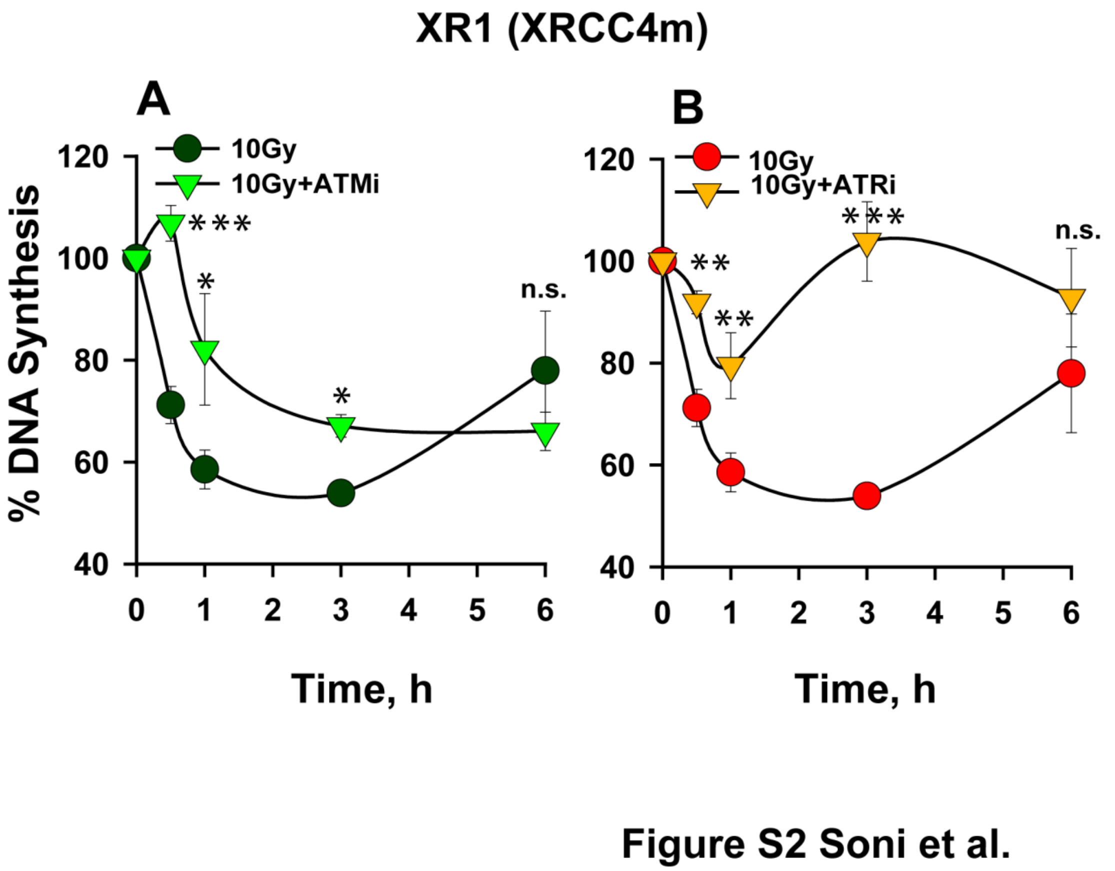

Figure S2. Impact of ATMi, and ATRi on IR-induced intra-S-phase checkpoint in XRCC4 mutant cells, XR1. (A) Percentage of DNA synthesis over unirradiated controls was measured after exposure to 10 Gy IR in the presence or absence of ATMi. (B) As in (A) in the presence or absence of ATRi. Error bars represent the standard deviation from 3 independent experiments. The significance of differences between individual measurements is indicated by * symbol: *$p < 0.05$, **$p < 0.01$, ***$p < 0.001$, n.s. non-significant.
